# The Importance of Amodal Completion in Everyday Perception

**DOI:** 10.1177/2041669518788887

**Published:** 2018-07-31

**Authors:** Bence Nanay

**Affiliations:** University of Antwerp, Belgium; Peterhouse, Cambridge University, UK

**Keywords:** attention, imagery, perceptual organization, scene perception

## Abstract

Amodal completion is the representation of those parts of the perceived object that we get no sensory stimulation from. In the case of vision, it is the representation of occluded parts of objects we see: When we see a cat behind a picket fence, our perceptual system represents those parts of the cat that are occluded by the picket fence. The aim of this piece is to argue that amodal completion plays a constitutive role in our everyday perception and trace the theoretical consequences of this claim.

## What Is Amodal Completion?

Amodal completion is the representation of those parts of the perceived object that we get no sensory stimulation from. In the case of vision, it is the representation of occluded parts of objects we see: When we see a cat behind a picket fence, our perceptual system represents those parts of the cat that are occluded by the picket fence.

We also get amodal completion in nonvisual sense modalities. In tactile perception, it is the completion of those parts of the objects we touch that are not in direct contact with our hand, for example. We complete those parts amodally.

In the case of audition, when we hear a loud bang while listening to a tune, the auditory system continues to represent the tune even in that brief moment when the bang is the only auditory stimulation. What we have here is a form of temporal occlusion, where the bang occludes part of the tune. A popular demonstration of auditory amodal completion is the American late night show host Jimmy Kimmel's segment “A week in unnecessary censorship,” where he beeps out completely harmless words from famous politicians, making them sound like expletives.

In the history of psychology, people often made a distinction between modal and amodal completion. Given that this distinction was made differently by different psychologists, this needs a bit of discussion (see Singh, 2004 for a good overview of some of the differences between modal and amodal completion).

The standard way of drawing this distinction is as follows (see, e.g., Tse, 1999, pp. 37–38). In the case of the amodal perception, we represent objects behind an occluder, whereas in the case of modal completion, we represent an object in front of inducers, as in the case of the Kanizsa triangle.

**Figure:**
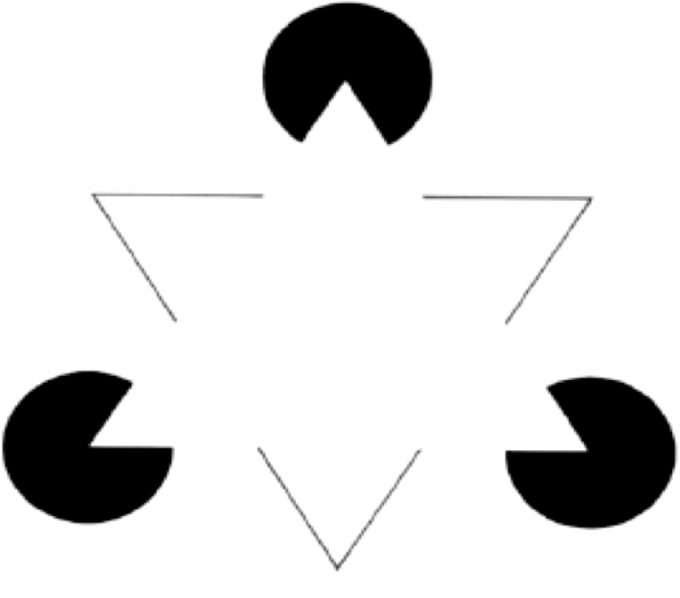

Albert Michotte, who introduced the term, however, drew the distinction in terms of phenomenology (Michotte & Burke, 1951). In the original Michotte account, the difference is that while in the case of modal completion we experience the illusory contour as a contour between two different colors, amodally completed contours are experienced without a perceived color difference. Unfortunately, often a much less sophisticated phenomenological difference is posited between modal and amodal completion, along the lines that while we consciously experience the modally completed contours, we do not consciously experience amodally completed contours (it should be added that Michotte himself was not always crystal clear about the nature of this phenomenological difference, see, e.g., Michotte, Thinés, & Crabbé, 1964). The Kanizsa triangle would count as modal completion on this approach as well but for a different reason: The completed contours are visually experienced as a contour between two different colors.

Neither of these ways of keeping apart modal and amodal completion is very convincing. The standard version is especially confusing as many instances of subjective contours fail to correspond either to something behind an occluder or something in front of inducers. Take the horse illusion, for example:

**Figure:**
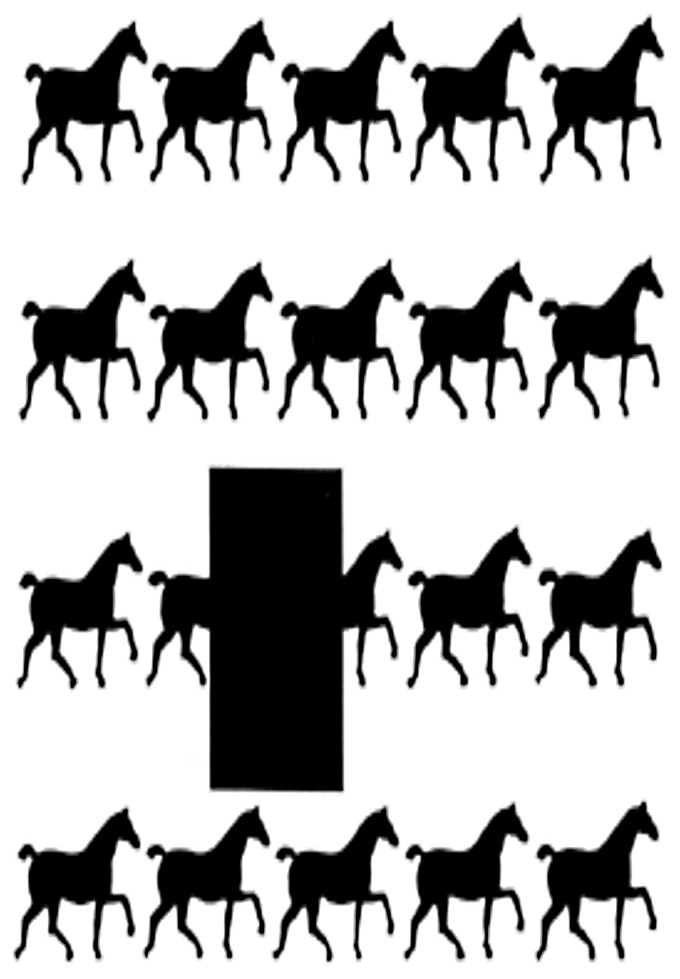

This is an illusion because many people visually represent a long horse where the black rectangle is. In that row, they see not five but four horses, one of which is a sausage horse. But would this count as amodal or modal completion according to the standard account? The horse is neither behind the rectangle nor in front of it. In fact, it could be seen as either—we could even intentionally switch these two possible visual interpretations if we want to. And many illusory contours work in the same way: They can be seen both as being occluded or being induced. In other words, the occluded or induced distinction is unlikely to be a very useful one when it comes to keeping apart modal and amodal completion (see also Michotte & Burke, 1951).

But how about the phenomenology-based distinction? I am skeptical of attributing too much importance to phenomenology in general and to a phenomenological way of drawing the modal versus amodal completion distinction in particular. One reason for skepticism is the significant interpersonal variations in amodal completion (see Sekuler & Palmer, 1992) and, more generally, in the vividness of nonretinal visual phenomena (Bergmann, Genc, Kohler, Singer, & Pearson, 2016; Cui, Jeter, Yang, Montague, & Eagleman, 2007; Dijkstra, Bosch, & van Gerven, 2017). I frequently give talks where I show the horse illusion to the audience, and each time the audience is divided whether they “see” a long horse. According to at least some versions of the phenomenology-based distinction, this would mean that some people in the audience complete modally, some others amodally. But this would make the distinction somewhat arbitrary.

The aim of this discussion is not to dismiss any distinction between modal and amodal completion (see Murray, Foxe, Javitt, & Foxe, 2004; Singh, 2004; Spehar & Halim, 2016). I raised these points because of the lack of clarity about just what counts as amodal completion. The deeper point here is that the neural mechanisms responsible for modal and amodal completion are the same. In both cases, we have shape completion already in the primary visual cortex (see Bakin, Nakayama, & Gilbert, 2000; Ban et al., 2013; Bushnell, Harding, Kosai & Pasupathy, 2011; Emmanouil & Ro, 2014; Hazenberg, Jongsma, Koning, & van Lier, 2014; Hedge', Fang, Murray, & Kersten, 2008; Komatsu, 2006; Kovacs, Vogels, & Orban, 1995; S. H. Lee et al., 2012; T. S. Lee & Nguyen, 2001; Lommertzen, Van Lier, & Meulenbroek, 2009; Pan et al., 2012; Scherzer & Ekroll, 2015; Shibata, Watanabe, Sasaki, Kawato, 2011; Smith & Muckli, 2010; Sugita, 1999; Vrins, De Wit, & Van Lier, 2009).^1^ In other words, from a neuroscience point of view, modal and amodal completion are very similar, if not equivalent (Davis & Driver, 1994; Driver, Davis, Russell, Turatto, & Freeman, 2001; Grossberg & Mingolla, 1985; He & Nakayama, 1992; Kellman & Shipley, 1991) . From this perspective, the difference between whether the subjective contour is conscious and unconscious and the difference between whether it is occluded or induced are superficial differences. As a result, I will treat them as superficial differences in what follows.

Amodal completion is not a perceptual curiosity: It is part of our ordinary perception. It happens very rarely in real-life situations that we can perceive an object without exercising amodal completion: In natural scenes, we always get occlusion because objects tend not to be fully transparent. Every time we see an object occluded by another object (which means every time we see anything in real life, barring odd cases of fully translucent visual scenes or very simple visual displays), we use amodal completion of the occluded parts of perceived objects (Bakin et al., 2000). And the same goes for the backside of any solid object—sometimes referred to as self-occlusion. Again, we do not receive any sensory stimulation that would correspond to the backside of solid three-dimensional objects, but there is nonetheless perceptual processing of this missing information—in a way reminiscent of amodal completion (Ekroll, Sayim, Van der Hallen, & Wagemans, 2016, Nanay, 2010a).

## What Kind of Mental State Is Amodal Perception?

When we see a cat behind the picket fence, we represent those parts of the cat that are occluded by the picket fence. The question is this: How do we do so? What kind of representations are the ones that we use in amodal completion?

There seem to be three straightforward answers (see also Kanizsa and Gerbino 1982):

- Amodal completion represents perceptually. Amodal completion is really perception as much as sensory stimulation-driven perception is.
- Amodal completion represents by means of beliefs or some other postperceptual mental states.
- Amodal completion represents by means of mental imagery.

I argue that empirical findings support (iii). (i) and (ii) are not empirically plausible ways of thinking about amodal completion (this is a different argument from the one I provide in Nanay, 2010a).

I do not take (i) to be a serious contender as we have no (local) sensory stimulation in amodal completion and we also lack any causal link with the represented feature—two extremely widely shared necessary conditions on perceiving.^2^ First, it has been very widely agreed on that perceiving implies having sensory stimulation. In the case of amodal completion, of course there is sensory stimulation—the perceptual system completes the figure amodally on the basis of this retinal stimulation. But there is no local retinal stimulation that would correspond to the amodally completed feature.

It should be emphasized that this claim is much weaker than saying that perception is object-involving or that it presupposes that the perceived object is in front of us and we are in perceptual contact with it. Even if there are no objects in front of us or the light that hits our retina fails to come from them, we can still have sensory stimulation—we can still have light (coming from *somewhere*) hitting our retina. It is this latter much weaker condition that I take to be necessary for perception.

Can one insist that perception does not require (local) sensory stimulation? Yes. J. J. Gibson, for example, did just that: He argued that amodal completion is genuinely perceptual. As he says, “the perception of occlusion, it seems to me, entails the perception of *something* which is *occluded*” (Gibson 1972, p. 229). Gibson's way of thinking about perception is very much against the mainstream in psychology. But there are some other, more interesting, dissenters. One interesting view in this context is Roy Sorensen's. Sorensen argues explicitly that sensory stimulation is not necessary for perception (Sorensen, 1999, 2007): Hearing silence, for example, is still hearing and it is different from being deaf. But even if we accept Sorensen's view, it would still not make amodal completion perceptual. He writes: “To see an object, the object must be causally responsible for the visual information” (Sorensen, 1999, p. 45). The cat's occluded tail is not causally responsible for any visual information; thus, we cannot represent it perceptually. So even if we deny that sensory stimulation is necessary for perception, unless we also deny some kind of causal link as necessary for perception, we can safely conclude that amodal completion does not represent perceptually.

But how does amodal completion represent then? The second option was that it represents by means of beliefs or some other postperceptual mental states. Here is a serious empirical problem with this proposal: We have plenty of evidence that amodal completion happens very early in perceptual processing. It is well-documented that there is early cortical processing in amodal completion and even processing already in the primary visual cortex (see Bakin et al., 2000; Ban et al., 2013; Bushnell et al., 2011; Emmanouil & Ro, 2014; Hazenberg et al., 2014; Hedge' et al., 2008; Komatsu, 2006; Kovacs et al., 1995; S. H. Lee et al., 2012; T. S. Lee & Nguyen, 2001; Lommertzen et al., 2009; Pan et al., 2012; Scherzer & Ekroll, 2015; Shibata et al., 2011; Smith & Muckli, 2010; Sugita, 1999; Vrins et al., 2009).

This should be a decisive objection to account (ii): If amodal completion happens in the primary visual cortex, it is not happening on the level of beliefs or nonperceptual representations—it happens much earlier. But the proponents of account (ii) have a somewhat desperate way of responding: They could say that the early cortical activation is not amodal completion—It is a consequence of the amodal completion that is done by beliefs. So the view proponents of account (ii) could argue, as a last resort, that amodally completed properties are represented by beliefs and this, in turn, activates the primary visual cortex by means of some kind of top-down influence.

There are two problems with this response: one conceptual and one empirical. First, the conceptual: This response would amount to saying that the retinotopic perception of the visible parts of the object gives rise to a nonretinotopic belief (the actual amodal completion), which then triggers, in a top-down manner, the retinotopic representation of the occluded parts of the object. So by representing the occluded part by means of a belief, we lose retinotopy, which then somehow gets put back in for the well-demonstrated retinotopic activation of the primary visual cortex. Not a very plausible picture.

Second, and perhaps more decisively, there is plenty of empirical evidence that this picture cannot be correct given what we know about the timing of amodal completion. Amodal completion in the early cortices happens within 100 to 200 milliseconds of retinal stimulation (Rauschenberger & Yantis, 2001; Sekuler & Palmer, 1992—this is true even of complex visual stimuli, like faces, see Chen, Liu, Chen, & Fang 2009, see also Lerner, Harel, & Malach, 2004; Rauschenberger, Liu, Slotnick, & Yantis, 2006; Yun, Hazenberg, & van Lier, 2018; for detailed studies that track the (very quick) temporal unfolding of amodal completion in different parts of the visual cortex). And this is much much shorter than the time that would be needed for perceptual processing to reach all the way up to beliefs or nonperceptual representations and then trickle all the way down again to the primary visual cortex (see Lamme & Roelfsema, 2000; Thorpe, Fize, & Marlot, 1996; for the temporal unfolding of visual processing in nonamodal cases). To sum up, account (ii) is not consistent with neuroimaging data about early cortical processing in amodal completion.

What other options do we have? My claim is that amodal completion is a form of mental imagery. Depending on one's concept of mental imagery, this claim may come across as pretty farfetched. So it is important to emphasize that what I mean by mental imagery is what psychologists and neuroscientists mean by mental imagery (which is often different from the way philosophers think of this concept). Here is a famous characterization of mental imagery by Kosslyn, Behrmann, and Jeannerod (1995):

Visual mental imagery is “seeing” in the absence of the appropriate immediate sensory input, auditory mental imagery is “hearing” in the absence of the immediate sensory input, and so on. Imagery is distinct from perception, which is the registration of physically present stimuli. (p. 1335)

And here is a more recent definition from a review article on mental imagery: “We use the term ‘mental imagery’ to refer to representations [ … ] of sensory information without a direct external stimulus” (Pearson, Naselaris, Holmes, & Kosslyn, 2015, p. 590). If we do a bit of touching up of these ways of characterizing mental imagery, we get the following definition: *Mental imagery is perceptual processing that is not triggered by corresponding sensory stimulation in a given sense modality* (see Nanay, 2015, 2016a, 2016b, 2018, in press).

Perceptual processing means early cortical processing. In the visual case, this definitely includes processing in V1, V2, V4/V8, MT. The earlier the processing is the more reason we have to consider it to be perceptual processing (and not postperceptual processing). This perceptual processing can be triggered by corresponding sensory stimulation. This happens in ordinary vision. But perceptual processing can also be triggered in a way that is not triggered by corresponding sensory stimulation. Perceptual processing of this kind is mental imagery. Whether the sensory stimulation and the early cortical processing really correspond could be easily determined given the retinotopy of the visual early cortical areas (Grill-Spector & Malach, 2004).

But things are more complicated when it comes to other aspects of perception, for example, color perception. We cannot just read off the correspondence or lack thereof off the relationship between the retina and V4. Colorblind people never have the kind of correspondence between the retina and V4 that we would expect—yet, they, presumably, should not be described as always entertaining mental imagery.

In order to bypass these worries, here is a more neutral way of characterizing correspondence. A certain sensory stimulation reliably triggers an early perceptual processing of a certain kind. If this early perceptual processing happens without sensory stimulation that would reliably trigger it, this means that there is no correspondence—we have mental imagery. It is important that this reliability is understood restricted to the subject in question. In a colorblind person, for example, a certain sensory stimulus would reliably trigger certain early perceptual processing in a way that would not be reliably triggered in other, not colorblind subjects.

Note that I do not use the terms “perception” and “perceptual processing” interchangeably. Perception is perceptual processing triggered by corresponding sensory stimulation in the relevant sense modality. But perceptual processing does not have to be triggered by corresponding sensory stimulation in the relevant sense modality—in the case of mental imagery, it is not.

Mental imagery in this sense does not have to be voluntary (and indeed many instances of mental imagery are not voluntary—think of involuntary flashbacks or earworms). And it does not have to be conscious either. Just as sensory stimulation-driven perceptual processing can be conscious or unconscious, the same is true of perceptual processing that is not triggered by corresponding sensory stimulation. Note that neither definitions I quoted above talk about consciousness or voluntariness. These are features of some subcases of mental imagery (ones philosophers tend to focus on), but not of all.

Amodal completion counts as mental imagery in this sense. It is early perceptual processing of a contour (as we have seen, processing already in V1) that is not triggered by corresponding sensory stimulation. When you amodally complete the cat behind the picket fence, in V1 there is activation of direction-sensitive neurons that would correspond to where the cat's outlines would be, but there is nothing on the retina that would correspond to these contours. So amodal completion is perceptual processing that is not triggered by corresponding sensory stimulation. It is mental imagery.

Amodal completion is, of course, in some sense, driven by the retinal image. What determines how the occluded parts of the cat are represented is the retinal stimulation of the nonoccluded parts. But the representation of the amodally completed features is not driven by the corresponding sensory stimulation (i.e., sensory stimulation that would correspond to the amodally completed features) for the simple reason that there is no corresponding sensory stimulation (i.e., there is not sensory stimulation that would correspond to the amodally completed features).

Not everyone uses the concept of mental imagery the way I outlined (and what I take to be the consensual view in psychology and neuroscience—but not in philosophy). Here is a puzzling remark by Vebjorn Ekroll et al. (2016) that may seem to suggest that not everyone is on board with the idea that amodal completion is a form of mental imagery:

Our experience of the hidden backsides of objects is sometimes based on genuine perceptual representations rather than mere cognitive guesswork or imagery, despite the lack of any direct sensory stimulation reaching the eye from the hidden backsides themselves. (p. 3)

This quote seems to present a choice between perceptual representation on one hand and cognitive guesswork and imagery on the other. In my account, imagery is a form of perceptual processing, so it is definitely on the perceptual side of this divide and has very little to do with cognitive guesswork. Ekroll seems to rely on a very odd way of understanding mental imagery, probably as active imagination. If we understood mental imagery this way then amodal completion is clearly not imagery. If we understood mental imagery as perceptual processing not triggered by corresponding sensory stimulation in the relevant sense modality, then amodal completion is clearly mental imagery.

Understanding amodal completion as a subspecies of mental imagery can also help us to understand the function of mental imagery better. Most uses of mental imagery are remarkably useless, so much so that one may wonder how this ability may have evolved. But amodal completion is very useful indeed: Spotting the lion's tail sticking out of the bush and amodally completing the rest of the lion is as important for survival as perceiving the lion.

## Varieties of Amodal Completion

Amodal completion comes in many varieties. First, it may be, but it does not have to be, sensitive to top-down influences (Hazenberg et al., 2014, Hazenberg & Van Lier, 2016; Lee & Vecera 2005, 2010, Van Lier & Gerbino 2015). Some instances of amodal completion may be fully bottom-up driven, like the completion of shapes purely on the basis of Gestalt forms (that can go against our best judgments). But some other times, amodal completion is driven in a top-down manner as in the case of seeing the cat behind the picket fence. Depending on what cats I have encountered before, the way I complete this figure would be very different. The same goes for the amodal completion of letters and words. Higher order knowledge and expectations play an important role here.

Here is an evocative example, taken from the 1980s classic comedy *Top Secret*. One of the many visual jokes of the film has the main character crawl in the mud, shown in close up and suddenly he faces two Nazi boots, framed in a way that we can only see the boots. He looks scared and the camera zooms out, revealing that it is only two boots standing in the mud, there is no Nazi officer in them. Again, we use amodal completion to represent what is outside the frame (a theme I will come back in the last section), and we use a lot of high-level information to complete what is outside the frame, for example, knowledge that Nazi boots usually continue upwards in Nazi officiers.^3^

Second, amodal completion may be conscious or unconscious. Given the sheer amount of amodal completion the visual system needs to do at any given moment, amodal completion is normally unconscious. When I see 50 cats behind the picket fence, I do not form conscious mental imagery of all occluded parts of all the fifty cats. But amodal completion can be conscious if, for example, we are really interested in some of the occluded features. If for some reason I need to attend to the left eye of one of these 50 cats and it is occluded by the fence, I am likely to represent this left eye consciously.

This takes us to a third, related, but different distinction: Amodal completion can be attended or unattended. I can shift my attention from one perceived objects to another and I can do the same when it comes to amodally completed parts of perceived objects. We can attend to some properties of amodally represented parts of perceived objects, but normally we ignore most of these properties (see also De Weerd, Smith, & Greenberg, 2006 for empirical support of this).

Further, most of the time you attribute very determinable properties to amodally represented parts of perceived objects (this point is not independent from the previous one: see Yeshurun & Carrasco 1998; Nanay, 2010b on the relation between attention and the attribution of determinate properties). But if you are really interested in them for some reason, you can attribute very determinate properties (determined at least partly in a top-down manner). If I see you with your hands in your pocket, I am unlikely to have a determinate representation of how you move your fingers in your pocket. But if I attend to this very thing, I may attribute more determinate properties to the whereabouts of each of your fingers (which would be determined [at least partly] in a top-down manner).

## Amodal Completion and Perceptual Justification

The picture we ended up with is one where what we take to be perception is really a mixture of two things: sensory stimulation-driven perception and amodal completion. And as amodal completion is a form of mental imagery, this means that everyday perception is a mixture between sensory stimulation-driven perception and mental imagery.

This conclusion is a version of the old and influential idea that imagination is “a necessary ingredient of perception itself” (Strawson, 1974, p. 54—the metaphor and the quote are originally from Kant (*Critique of Pure Reason*, A120, fn. a, see also Sellars, 1978), but it has become a widespread slogan. Eugène Delacroix, for example, wrote: “Even when we look at nature, our imagination constructs the picture.”^4^ Mental imagery is different from imagination and I am not sure that imagination is in fact a necessary ingredient of perception, but as long as amodal completion counts as mental imagery, we can conclude that mental imagery is a necessary ingredient of perception itself.

In some very rare examples of simple two-dimensional visual displays, there is no amodal completion (there may still be some other forms of mental imagery, e.g., multimodal mental imagery, involved; see Nanay, 2018). In these cases, there is only sensory stimulation-driven perception. But these cases are extremely rare in everyday perception. In the vast majority of everyday perceptual scenarios, amodal completion is present and it gets combined with the sensory stimulus-driven perceptual processing.

This has serious consequences for epistemology (see also Chudnoff, 2018a, 2018b). Again, in the vast majority of everyday perceptual scenarios, what we take ourselves to perceive we at least partly represent by means of amodal completion. But as at least some of these episodes of amodal completion are subject to top-down influences, perception per se can also be subject to top-down influences. This argument for top-down influences on perceptual processing is different from the standard philosophical ones (Macpherson, 2012; Siegel, 2011; Stokes, 2012) and it is not susceptible to some of the criticism they face (see, e.g., Firestone & Scholl, 2014, 2016; Pylyshyn, 1999, see also Teufel & Nanay 2017).

If perception is sensitive to top-down influences because of the top-down sensitivity of amodal completion, then it is not an unbiased way of learning about the world, as our preexisting thoughts, beliefs and expectations could influence how and what we perceive (see Siegel, 2011 on a version of this worry). So we get a form of vicious circularity: Our beliefs, thoughts and expectations are supposed to be based on and justified by our perceptual states, but these perceptual states themselves are influenced by our beliefs, thoughts and expectations (because of the top-down influences on perception via amodal completion).

But I want to argue that the role amodal completion plays in everyday perception poses an even more significant worry for epistemology, regardless of whether it is influenced in a top-down manner.

Knowledge is supposed to be a good thing because it tracks truth. And perception is supposed to be a good way of acquiring knowledge because perception tracks truth. Sensory stimulation-driven perception does indeed track what it is of. Light from the perceived object hits our retina and that is the sensory stimulation that gets processed. But amodal completion is by definition not sensory stimulation driven. So it is cut off from the object it is about. One link in the causal chain is missing.

When we see a cat behind a picket fence, what is supposed to do epistemic work is not the perception of the unoccluded slices of the cat but rather the perception of the entire cat. But the perception of the entire cat is the result of both stimulus-driven perception (of the unoccluded slices of the cat) and mental imagery (of everything else). So whatever is supposed to do the epistemic heavy-lifting is partly constituted by mental imagery—something not particularly well-suited at all for any epistemic role as it fails to track the way the world is. If it is true that the vast majority of perceptual states are mixed perception/mental imagery cases, then almost all instances of perception are mixtures of a state that is supposed to track the truth (perception) and a state that, on the face of it, is not (mental imagery).

I said that amodal completion is, by definition, a step removed from the truth it is supposed to track. Of course it can track truth albeit in a fallible manner. It can be fooled, but in the vast majority of cases it is not. Nonetheless, whether perception can justify beliefs depends on empirical facts about the reliability of the mechanisms of amodal completion involved in perception.

We can make the default assumption that our perceptual system built pretty good mechanisms for amodal completion on the basis of local and global contextual information that does co-vary with the scene in front of us. But this is enough to resist dogmatism about perceptual justification.

Dogmatism is the philosophical view that “whenever you have an experience as of p, you thereby have immediate prima facie justification for believing p” (Pryor, 2000, p. 536). Similarly, “when it seems as if *P* and there is no evidence to the contrary, it is *reasonable to believe P*” (Huemer, 2001, p. 103—Huemer calls the view “conservativism”). The second quote continues with the assertion that this “is a necessary truth, not a contingent one” (Huemer, 2001). Although according to dogmatism, it is true in all possible worlds that “when it seems as if *P* and there is no evidence to the contrary, it is *reasonable to believe P*,” if my argument is correct, then it is false even in the actual world. It should also be noted that dogmatism is especially problematic in the light of recent findings that we treat amodally completed features as more reliable then features that are not amodally completed (Ehinger, Hausser, Ossandon, & Konig, 2017).

A lot more work needs to be done in order to show that we are justified to move from (amodal completion-tinted) perception to belief. Again, this is not to say that we cannot eventually do so, we surely can. But any such move is very far away from a “prima facie” or “immediate” justification the dogmatists want.^5^ It would need to involve a close empirical examination of the reliability of the processes that give rise to amodal completion.

The conclusion is that the question of perceptual justification is at least in part an empirical question—it requires the examination of the reliability of the kinds of amodal completion that play a constitutive part of perception per se.

## Amodal Completion in the Visual Arts

In closing, I want to examine a less obvious role amodal completion plays in our life and it is our engagement with visual artworks.

One (somewhat obvious) way in which amodal completion plays a role in our engagement with visual arts follows from the simple fact that most pictorial art does not encompass the entire visual field. So those parts of the depicted scene that fall outside the frame could be, and very often are, represented by means of amodal completion.

Some artists very explicitly try to evoke our amodal completion of aesthetically relevant properties outside the frame. One famous example would be Degas, who likes to place the protagonists of her paintings in a way that only parts of them are inside the frame. The rest we need to complete by means of amodal completion. In some extreme cases (e.g., Dancers climbing the stairs, 1886–1890, Musee D'Orsay), we only see someone's arm or the top of their head and we need to complete those parts of her body that are outside the frame amodally.

Jean-Luc Godard uses this way of composing images in an even more radical manner—in the films he made in the mid-1960s, many of the compositions are almost completely empty, with the face of the protagonist at the edge of the screen. Buster Keaton also uses the off-screen space but normally for comical effects. One example is the first shot of his short film *Cops* (1922), where we see the protagonist in close up behind bars and looking depressed—prompting the viewer to amodally complete the space outside the frame as a prison cell. But the second shot reveals that he is behind an iron garden gate taking to a girl who does not love him back. But off-screen space plays an aesthetically relevant role in many other films—for example, films by Jean Renoir, Ozu, Antonioni, and Bresson (see Burch, 1973, pp. 17–31).

Amodal completion can play an important role within the frame as well. One classic example is Bunuel's *Belle de Jour*, where the Chinese businessman shows a little box to the Cathrine Denevue character, who is clearly fascinated by what is inside. She sees it, he sees it, but we, the viewers do not. There is a humming voice coming from the box, but we never see what is inside. We have a very indeterminate amodal completion of what could possibly be in the box. And it is aesthetically relevant that the property that we thereby represent by means of amodal completion is indeterminate. It is not that we are not smart enough to find out what it is—whatever is in the box is left intentionally indeterminate.

Robert Bresson often uses amodal completion this indeterminate way, so much so that he even takes this use of amodal completion to be the mark of a “good” director (or, as he would put it, of a cinematographer, not merely of a director): “Don't show all sides of the object. A margin of indefiniteness” (Bresson, 1975/1977, p. 52).

One relatively simple use of amodal completion inside the frame comes from the abundance of occlusion in most everyday perceptual scenes (and, as a result, in most depicted scenes). Hiding aesthetically relevant properties in occluded parts of depicted objects has a long history, Rogier Van der Weyden plays with this in his *Seven Sacraments* (Antwerp), where he depicts one of the characters in a way that only the tip of his nose and chin are visible. Antonioni's *L'Eclisse* (1962) uses occlusion in a way that is clearly aesthetically relevant—for example, when we first see the two protagonists in the same frame both half occluded by the same giant column. And Godard's *Vivre sa Vie* (1962) starts with a long (7 minute) scene where the two main protagonists are filmed from behind—we hear their conversation, but we do not see their face. We need to use amodal completion to represent very important aesthetically relevant properties.

Some less high-brow examples: Monty Python's *How not to be seen* sketch relies entirely on the comic effects of amodal completion we use to represent occluded people. Also, in the sitcom *Seinfeld*, one of the recurring characters, Mr. Steinbrenner is only ever shown occluded. We sometimes see his head from behind, but his face is occluded. When we first see him, we only see his hand when he shakes hand with George, but the rest of his body is occluded behind a wall. And we sometimes see the shadow of his profile, but the only way we can represent his face is by means of amodal completion.

In the *Seinfeld* example (and in the Godard example as well), the use of occlusion is really a game or a running (visual) gag. But it can also be used in a more disconcerting manner, where the occluded parts of the scene are represented as something that is potentially dangerous or uncertain. Marguarite Duras's *India Song* (1975) is a clear example, where the vast majority of the shots hide a large occluded space, typically another room, in the background, where something potentially important could be happening, but we never see what that is. Rene Margritte's paintings and Andres Serrano's photographs almost always hide some aesthetically relevant features behind an occluder in a way that we can only form very indeterminate mental imagery of what is occluded. And Apichatpong Weerasethakul's films use this effect as the general emotional background that creates a sense of anxiety because we have no idea what is hidden behind, say, the jungle foliage in *Tropical malady* (2004).

Again, this, unlike the *Seinfeld* example, saddles us with deliberately indeterminate amodal completion. There is, presumably, a fact of the matter about how Mr. Steinbrenner looks (and the same goes for how Nana looks in Godard's *Vivre sa Vie*). But there is no fact of the matter about what is in the box in *Belle de Jour* or in the next room in *India Song* or in the jungle in Apichatpong Weerasethakul's films. And this is what makes these aesthetically relevant properties that are represented by means of indeterminate mental imagery disconcerting. As Proust says, “It's so soothing to be able to form a clear picture of things in one's mind. What is really terrible is what one cannot imagine” (Proust, 1913/1928, p. 525).

## Notes

1. See also the emerging research on eye movements in modal and amodal completion (De Grave, Hesse, Brouwer, & Franz, 2008; Desanghere & Marotta, 2015; Plomp, Nakatani, Bonnarde, & van Leeuwen, 2004; Vishwanath & Kowler, 2004; Troncoso, Macknik & Martinez-Conde, 2008)

2. And not just on veridical perception as there needs to be some kind of causal link in the case of illusory perception (something often highlighted when contrasting illusion and hallucination).

3. Robert Briscoe (Briscoe, 2011) argues (against Nanay, 2010a) that amodal completion is not a monolithic phenomenon—it comprises what he calls NA completion and C completion. NA completion is fully bottom‐up, whereas C completion is top‐down. I fully agree that amodal completion comes in many varieties. Some are top‐down, some are bottom‐up. And there are differences in how early or how late the involved perceptual processing is. But they all have a common denominator, namely they consist of perceptual processing not triggered by sensory stimulation in the relevant sense modality. This is true both of what Briscoe calls NA completion and of what he calls C completion. So the disagreement between Briscoe and me is really a terminological one.

4. Delacroix: *Journal*, 1859, September 1.

5. It needs to be acknowledged that dogmatists are internalists about justification: They think that whether or not my belief is justified depends only on what I am aware of (or, according to some weaker versions of internalism, on what is in my head). So, strictly speaking, dogmatists could just deny that the reliability of perception has anything to do with perceptual justification at all. I don’t think that this would be a good option for the dogmatist, but I can’t argue for this claim here. For a full exposition of this argument, see Helton and Nanay (forthcoming).
